# Association between Dietary Habits and Parental Health with Obesity among Children with Precocious Puberty

**DOI:** 10.3390/children7110220

**Published:** 2020-11-08

**Authors:** Yong Hee Hong, Yeon Ju Woo, Jong Hyun Lee, Young-Lim Shin, Hee-Sook Lim

**Affiliations:** 1 Department of Pediatrics, Soonchunhyang University Bucheon Hospital, Soonchunhyang University School of Medicine, Bucheon 14584, Korea; hongyonghee@schmc.ac.kr (Y.H.H.); 121738@schmc.ac.kr (Y.J.W.); ylshin@schmc.ac.kr (Y.-L.S.); 2 Department of Pediatrics, Soonchunhyang University Gumi Hospital, Soonchunhyang University School of Medicine, Gumi 39371, Korea; ljh4722@schmc.ac.kr; 3 Department of Food Sciences and Nutrition, Yeonsung University, Anyang 14011, Korea

**Keywords:** precocious puberty, obesity, parent child relations, body image, eating behavior

## Abstract

Precocious puberty, resulting in various physical, mental, and social changes, may have negative consequences for children and their families. In this study, we investigated whether there were differences between parental obesity, children’s and parent’s awareness of body shape, and dietary habits according to obesity levels in children with precocious puberty. A total of 193 children (93.3% girls) diagnosed with precocious puberty were classified into three groups according to their obesity levels. Negative body shape awareness and dissatisfaction were significantly higher in the obese group than in the normal-weight group, and parents were more likely to perceive their children as fat than the children themselves. In addition, the obesity rate of parents in the obese group was higher, and the body mass indexes of children and parents were significantly correlated. The nutrition quotients (NQs) were revealed to be significantly lower in the obese group with significantly lower scores. The significant factors related to obesity were the awareness of a fatty body image and dissatisfaction, obesity of the parents, and the NQ. The results show that obese children with precocious puberty were more vulnerable to negative lifestyle, family environment, and self-esteem effects than their normal-weight peers. Therefore, various interventions, such as environmental management, psychological support, and nutrition education, are needed that focus on the obesity and health conditions of children with precocious puberty.

## 1. Introduction

Precocious puberty is defined as the onset of secondary sexual characteristics before the age of 8 years in females and 9 years in males [1]. It is categorized as central or peripheral precocious puberty depending on the primary cause; central (also known as true) precocious puberty is a result of premature activation of the hypothalamic–pituitary–gonadal axis. It occurs more frequently in girls than boys, and most cases of precocious puberty in girls are idiopathic. In boys, on the other hand, precocious puberty is less common but, when it occurs, potentially dangerous structural central nervous system abnormalities are more common than in girls [2].

Several possible causes for the recent rapid increase in the reported incidences of precocious puberty and medical treatment for it have been proposed, including increases in childhood obesity, opportunities to exposure to sexual stimulation through TV and the Internet, environmental hormones caused by environmental pollution, and parental focus on child development [3,4]. Children with precocious puberty reportedly experience emotional confusion due to the mismatch between their mental and physical development and the associated social problems such as exposure to adolescent sexual violence, premature pregnancy, and deviant behavior [5]. One reason for the rapid increase in precocious puberty is the consumption of high-calorie, high-fat foods; these habits are also closely related with childhood obesity. Additionally, hormone production may be influenced by obesity due to overeating, stress, and environmental factors [4,5,6].

Among the various factors that may lead to precocious puberty, nutritional status and body fat mass are especially important. Nutrition during prepuberty, and even during infancy and childhood, has a significant impact on pubertal development, and several studies have reported that pubertal development is more rapid in obese children [5,6,7]. Obesity may contribute to accelerated secondary sexual development and growth in girls by increasing estrogen levels and androgen aromatization [8]. Socioeconomic growth and the improvement of health status in Korea have tended to accelerate puberty, and the number of children with precocious puberty is rapidly increasing [9]. However, although many studies have focused on the relationship between obesity or dietary habits and precocious puberty, relatively few studies have examined the dietary habits of patients with precocious puberty and their families.

Our study analyzed differences in family composition, parental obesity status and chronic disease status, and dietary habits according to the obesity level of children with precocious puberty. This study aimed to evaluate environmental and dietary factors that affect obesity in children with precocious puberty and to identify key points in health management.

## 2. Materials and Methods

### 2.1. Study Subjects

The subjects of this study were patients who visited the Department of Pediatrics, Soonchunhyang University, Bucheon Hospital, and Soonchunhyang University, Gumi Hospital, for treatment of central precocious puberty from 1 January 2018 to 30 July 2019. Central precocious puberty was diagnosed when secondary sexual characteristics appeared before the age of 8 years in girls and 9 years in boys, bone age was advanced for the patient’s chronological age, and the maximum luteinizing hormone level was higher than 5 IU/L in a Gonadotropin-releasing hormone (GnRH) stimulation test [10]. Patients with precocious puberty who were found to have underlying causes, such as brain tumors, thyroid abnormalities, ovarian tumors, or other disease, were excluded. Only children and caregivers who agreed to participate in the study were included as subjects. A survey was conducted including 249 children and their caregivers who had consented to the use of their medical records. Among them, 54 patients who provided incomplete responses and two who were diagnosed in other hospitals, were excluded. The final sample included a total of 193 patients. This study was approved by the Institutional Review Board of Soonchunhyang University, Bucheon Hospital (IRB number 2017-07-004). Written informed consent was obtained from all patients. All subjects voluntarily agreed to participate in the study after receiving a detailed description of the procedures and goals. The study protocol conformed to the ethical guidelines of the World Medical Association’s Declaration of Helsinki.

### 2.2. General Characteristics, Anthropometric Measurements, Blood Test, and Nutrient Quotient Analysis

Anthropometric data, including age, height, and weight, were acquired from medical records at the time of diagnosis. Height was measured at the outpatient department by a single skilled staff member using a portable height measuring bar with the patient wearing light clothing. Body weight was measured using an electronic scale and recorded in kilograms. Body mass index (BMI, kg/m^2^) was calculated using the measured height and weight, and BMI z-scores, adjusted for the influence of age and gender, were calculated based on the 2017 Korean national growth charts for children and adolescents provided by the Korea Centers for Disease Control and Prevention [11]. Subjects were classified into three groups according to the BMI percentile based on recommendations from a committee of experts from the American Academy of Pediatrics expert: normal, overweight, and obese [12]. Overweight was defined as children with a BMI in the 85–94th percentile for age and sex, and obese was classified as those in the 95th percentile and above. Blood test results, performed at diagnosis, reviewed by a retrospective survey of medical records included aspartate aminotransferase (AST), alanine aminotransaminase (ALT), glucose, and total cholesterol for the screening of the comorbidities.

Other variables assessed by the questionnaire included family composition and socioeconomic status, subjective awareness and satisfaction of body shape, parents’ health status, and dietary quality and food behavior. The composition of the family, the number of siblings, the family’s economic level by Korea’s health insurance criteria (i.e., high, middle–high, middle, and middle–low), and the parents’ educational level (i.e., ≥college, ≤high school) were investigated. Awareness of and satisfaction with a child’s body shape by both the child and their parents has been studied in previous research [13,14]. After calculating the BMI values of the father and mother based on their anthropometric information, the parents were also classified using the BMI (normal: 18.5–22.9 kg/m^2^; overweight: ≥23.0 and ≤24.9 kg/m^2^; obesity: ≥25.0 kg/m^2^). Chronic diseases (hypertension, diabetes, and dyslipidemia) of the parents were also recorded.

The nutrition quotients (NQs) for children developed by the Korean Nutrition Society is widely used in nutrition research with Korean children, as it can comprehensively evaluate children’s nutritional status by examining their dietary quality, nutrient intake, and dietary behavior through a survey [15,16,17]. A total of 19 assessment items are divided into five factors: balance, diversity, moderation, regularity, and practice. Those five factors are then further divided into questions on datary intake and behavior: balance (5 questions: intake frequency of whole grains, fruits, white milk, eggs, and legumes); diversity (3 questions: intake frequency of colorful vegetables, kimchi, and diverse side dishes); moderation (5 questions: intake frequency of fast foods, sweet foods, instant noodles, eating at night, and street foods), regularity (3 questions: meal regularity, eating breakfast, and exercise); practice (3 questions: hand washing, chewing well, and checking nutrition labels) [18]. The NQ total scores were categorized into five grades based on points out of 100, and nutritional status was divided into five grades: the highest (NQ ≥ 80.9), higher (73.8 ≤ NQ < 80.9), medium (56.5 ≤ NQ < 73.8), lower (47.6 ≤ NQ <56.5), and the lowest grade (NQ < 47.6) [12].

### 2.3. Statistical Analyses

All analyses were carried out using R (version 3.6.3; The R Foundation for Statistical Computing, Vienna, Austria), and a two-sided *p*-value of less than 0.05 was considered to indicate statistical significance. The demographic characteristics and clinical factors of the subjects are presented as means ± standard deviation for continuous variables and as frequencies with percentages for categorical variables. Wilcoxon’s rank-sum test was used for comparing the continuous variables, and the χ^2^ test or Fisher’s exact test were used for comparing the categorical variables as appropriate. The associations between the BMI of the children and the parents were evaluated by Pearson’s correlation coefficient analysis. Subsequently, multinomial logistic regression analyses were performed to evaluate factors affecting obesity status.

## 3. Results

### 3.1. Relationship between Anthropometric Status and Biochemical Data among Three BMI Groups

Subjects’ anthropometric and biochemical data are shown in Table 1. All subjects were classified according to their BMI percentile as follows: 135 of the 193 subjects were included in the normal-weight group (69.9%), 31 in the overweight group (16.1%), and 27 subjects were included in the obese group (14.0%). Of the 193 children, 13 were boys (6.7%) and 180 were girls (93.3%). The number of girls was significantly higher in all three groups (*p* = 0.028); the ratio of males to females was higher in the overweight and obesity groups than in the normal group, but the difference was not statistically significant. From the various blood tests performed, four parameters (i.e., AST, ALT, serum glucose, total cholesterol) were collected and analyzed in this study. The majority of the results were within a normal range, but, in the obesity group, some values for total cholesterol were in the “above normal” and “significantly higher than normal” ranges; the mean was 186.4 ± 26.4 mg/dL (*p* = 0.019). An ALT level was significantly higher in the obesity group than in the normal group (*p* = 0.005), but both groups remained in the normal range.

### 3.2. Comparison of Family Composition and Socioeconomic Status among the Three Groups

The comparisons of family composition and socioeconomic status among the three groups are shown in Table 2. There were no significant differences in the composition of the families and the number of siblings among the three groups. The most common educational level was represented by families in which both parents were college graduates; there was no significant difference among the three groups.

### 3.3. Comparison of Body Awareness and Parents’ Health Status among the Three Groups

**Figure 1:**
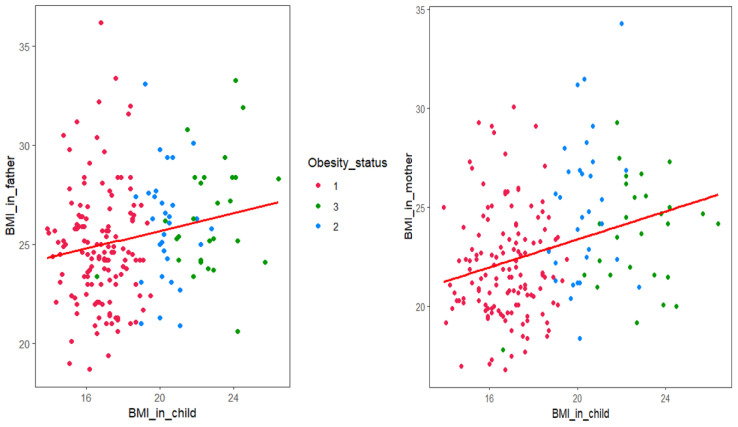
Correlation between children’s body mass index (BMI) z-score and parents’ BMI. 1, normal group; 2, overweight group; 3, obesity group. The father’s BMI showed a significant correlation with the child’s BMI (*r* = 0.199, *p* = 0.006). The mother’s BMI showed a significant correlation with the child’s BMI (*r* = 0.298, *p* < 0.001).

The comparisons of body cognition and parents’ health status are shown in Table 3 and Figure 1. When asked to describe their children’s bodies, the proportion of children and parents responding that they were fatty or very fatty was significantly higher in the overweight and obese groups than in the normal group (*p* < 0.001). An analysis of children’s body satisfaction revealed that the proportion who were “satisfied” or “very satisfied” was 46.2%, but satisfaction was significantly lower in the overweight group (*p* < 0.001). The parents’ responses showed that 39.9% were “satisfied” or “very satisfied” with their children’s bodies, a lower percentage than among the children. In the overweight and obese group, the satisfaction rate was significantly lower (*p* < 0.001). As a result of analyzing the responses and matching the rates of parents’ and children’s body shape awareness and satisfaction, disagreement in body perception rates was significantly higher in the obese group (*p* = 0.004). The mean BMI of the fathers was 25.3 ± 3.0 kg/m^2^ and was highest among fathers of those in the overweight group (*p* = 0.014). There was no significant difference in the percentage of fathers with chronic diseases among the three groups, but fathers of children in the obese group had the highest rate of such diseases. The mean BMI of the mothers was 22.8 ± 2.8 kg/m^2^ and was the highest among mothers of those in the overweight group (*p* < 0.001). The percentage of mothers with chronic diseases was lower than that of fathers, but similar to fathers; this percentage was highest in mothers of children in the obese group. The rate of obesity for both fathers and mothers was significantly higher in the overweight group, at 41.9% (*p* = 0.001). Analysis of the correlation between the children’s BMI z-scores and parents’ BMI revealed significant positive correlations for both mothers (*r* = 0.199, *p* = 0.006) and fathers (*r* = 0.298, *p* < 0.001).

### 3.4. Analysis of Nutrition Quotients (NQs) According to Obesity Status

The NQ scores, which reflect a comprehensive evaluation of children’s nutrition status and eating habits, are shown in Table 4. The overall mean NQ score was 60.3 ± 6.6; the score in the obese group was significantly lower than those for the normal and overweight groups (*p* < 0.001). When the factors that make up the scale were considered separately, the score for moderation was the lowest overall, at 45.9 ± 3.7, and was regularly the highest at 66.8 ± 5.6 points. The scores for each factor were significantly lower in the obese group than in the normal and overweight groups. The lowest factor score was for moderation in the obese group, at 42.5 ± 3.6.

### 3.5. Factors Affecting the Obesity Status for Children with Precocious Puberty

The logistic regression analysis results for factors affecting obesity status are shown in Table 5. When both children and parents judged that the body type was fatty or very fatty, the adjusted odds ratio (OR) was significantly higher (children, OR for fatty = 1.60; 95% confidence interval (CI): 0.98–2.52; OR for very fatty = 2.13; 95% CI: 1.44–2.88; parents, OR for fatty = 2.04; 95% CI: 1.01–2.49; OR for very fatty = 2.21; 95% CI: 1.56–3.23), and this tendency was similar for satisfaction of body shape. Children who were not satisfied at all with their body shape had a higher OR for obesity. In addition, the obese status of the father or mother was more related to the obesity of the child in the case of overweight or obese than normal weight. (Father, OR for overweight = 1.67; 95% CI: 0.99–1.62; OR for obesity = 2.53; 95% CI: 2.01–3.65; mother, OR for overweight = 1.21; 95% CI: 0.93–1.84; OR for obesity = 1.86; 95% CI: 1.35–2.29.) A lower NQ was significantly affected by the obese status of children, especially diversity, moderation, and practice.

## 4. Discussion

In this study, the proportion of girls showing precocious puberty was higher than that of boys, and the percentage of overweight was 16.1% and that of obesity was 14.0%. These values are similar to previous reports indicating that the incidence of precocious puberty is more than 10 times higher in females than in male children and that early puberty was more common in obese children [19,20]. Precocious puberty is associated with central obesity, and, although patterns of fat distribution are an important factor in predicting metabolic and cardiovascular diseases, this study did not provide useful information about fat distribution due to the limited indicators of obesity [21]. Precocious puberty has been reported to cause anxiety due to the physical changes, dissatisfaction with changes in body shape, loneliness, and other negative psychological effects [22]. Parents who are aware of their children’s physical changes also experience anxiety and, as parents, feel the economic difficulties in drug treatment [23,24]. In this study, the results regarding body shape awareness and satisfaction showed that children’s dissatisfaction with overweight or obesity was lower than the results reported by Cho et al. [25]. A notable result was that parents’ negative perceptions or dissatisfaction were greater than that of their children. Although the fundamental cause of this could not be determined, it is generally considered that parents’ concern about their children’s symptoms and prognosis also affected the children’s body shape awareness or satisfaction. Prior studies have shown that the parents of children with chronic diseases experience the children’s psychological burden of anxiety, fear, etc., and are more likely to experience depression and psychological stress due to their role as parents [26,27]. In children with precocious puberty, the better the parental communication and intimacy with the child, the lower the anxiety. Conversely, the less affection from the parent, the more the child’s behavior is likely to become negative [28]. Interaction with parents can have a significant impact on the emotional, social, and psychological experiences of precocious children, so it is important to encourage parents to recognize the importance of a more positive assessment and emotional interaction with children.

Among the causes of childhood obesity, eating habits and lifestyles are modifiable, and maintaining a balanced diet at home and receiving proper nutrition education are most effective during childhood when eating habits are formed [29]. In particular, children show a positive attitude toward good eating habits and can change their behavior by learning and acquiring nutrition-related knowledge. Studies have shown that obese children with precocious puberty have inappropriate eating habits and lifestyles such as short mealtimes, frequent beverages, lack of exercise, and prolonged TV viewing [30,31]. In this study, NQs, a proven nutrition assessment tool developed for Korean children, were used to analyze subjects’ food behaviors and meal quality comprehensively. The average score, 60.3 of 100 points, was considered a medium grade, and the obese group received a lower grade. Comparing these results with those from other domestic studies, the NQ total scores of these subjects were among the lowest reported, although the score for the practice factor was lower in a general population [12,32]. This study was the first to analyze NQs for children with precocious puberty, and there are many limitations to comparing them with NQs of general children in the previous study. However, it has been confirmed that the factors affecting obesity were NQ, diversity, moderation, and practice, so more active nutritional management is needed.

It is understood that the score for moderation was relatively low due to the high frequency of eating sweets, fast food, instant noodles, late-night snacks, and street foods and the children’s inability to control their consumption of these foods. Pearson et al. [33] found that students who reported a high frequency of eating snacks and fast foods exhibited low consumption of fruits and vegetables, and the consumption of such foods affected their intake at regular meals. Recently, nutrition problems have emerged among Korean children as a result of increased intake of high-calorie, low-nutrient foods, high volumes of sugars, and excessive consumption of ingredients such as caffeine and food additives [34,35]. Because children’s eating behavior is affected by family environmental variables, eating habits that contribute to their parents’ obesity and disease-inducing behaviors may also be related. More careful observation and management of healthy dietary habits for children with precocious puberty are needed, especially considering the poor NQ results among obese members of this group. In addition, it is considered that various nutrition education programs could induce desirable physical changes to enhance self-esteem and help to delay the progress of precocious puberty.

This study had several limitations. First, it is not possible to generalize the results of this study, as it was conducted with a limited audience in a single institution. In addition, the results reflect subjective evaluations because the data were collected through a self-report questionnaire. However, unlike previous studies, it is meaningful that the evaluation of body image was analyzed according to obesity degrees in children with precocious puberty, the relationship with parental variables was investigated, and the problems with eating habits were identified. Through this, it was possible to suggest a direction for improving the health of children with precocious puberty and to emphasize the importance of the parental role.

## 5. Conclusions

Obese children with precocious puberty had higher negative awareness and dissatisfaction with their body shape and reported inappropriate dietary habits. Family environmental factors as well as parents’ interest and support are very important if children with precocious puberty are to grow into healthy adults, and various interventions concerning social emotional stability and lifestyle management should be maintained.

## Figures and Tables

**Table 1:** Anthropometric status and biochemical data according to obesity status.

Variables	Total (*n* = 193)	Normal (*n* = 135)	Overweight (*n* = 31)	Obesity (*n* = 27)	*p*-Value
Sex					0.028
Boy	13 (6.7)	5 (3.7)	5 (16.1)	3 (11.1)	
Girl	180 (93.3)	130 (96.3)	26 (83.9)	24 (88.9)	
Height (cm)	134.01 ± 5.5	133.3 ± 4.8	135.0 ± 6.8	136.6 ± 6.2	0.002 ^a^
Weight (kg)	32.8 ± 6.3	29.9 ± 3.5	37.2 ± 5.2	42.6 ± 6.0	<0.001 ^a,b,c^
BMI (kg/m^2^)	18.2 ± 2.6	16.8 ± 1.4	20.3 ± 1.0	22.7 ± 1.9	<0.001 ^a,b,c^
BMI z-score	0.44 ± 1.06	–0.13 ± 0.66	1.33 ± 0.17	2.15 ± 0.62	<0.001 ^a,b,c^
AST (mg/dL)	23.3 ± 4.5	23.4 ± 4.5	23.5 ± 4.4	22.3 ± 4.6	0.448
ALT (mg/dL)	14.3 ± 6.2	13.5 ± 5.4	15.8 ± 6.0	16.7 ± 9.2	0.005 ^a^
Glucose (mg/dL)	99.1 ± 13.3	99.7 ± 14.3	96.2 ± 8.0	99.1 ± 13.1	0.639
Total cholesterol (mg/dL)	175.4 ± 27.7	172.8 ± 28.0	177.4 ± 25.3	186.4 ± 26.4	0.019 ^a^

BMI, body mass index; AST, aspartate aminotransferase; ALT, alanine aminotransferase. The data are presented as number (%) or the mean ± standard deviation. *p*-Value determined by analysis of variance test (continuous variables) or χ^2^ test (categorical variables). ^a^ There existed a significant difference between the normal and obesity groups. ^b^ There existed a significant difference between the normal and overweight groups. ^c^ There existed a significant difference between the overweight and obesity groups.

**Table 2:** Family composition and socioeconomic status according to obesity status.

Variables	Total (*n* = 193)	Normal (*n* = 135)	Overweight (*n* = 31)	Obesity (*n* = 27)	*p*-Value
Current composition					0.730
Grandparents + Parents	67 (34.7)	44 (32.6)	11 (35.5)	12 (44.4)	
Parents	121 (62.7)	87 (64.4)	19 (61.3)	15 (55.6)	
Grandparents + Parents + Others (aunts, uncle)	5 (2.6)	4 (3.0)	1 (3.2)	0 (0.0)	
Numbers of sibling					0.261
(include of child)					
1	36 (18.8)	27 (20.1)	8 (25.8)	1 (3.7)	
2	123 (64.1)	83 (61.9)	17 (54.8)	23 (85.2)	
3	32 (16.7)	23 (17.2)	6 (19.4)	3 (11.1)	
4	1 (0.5)	1 (0.7)	0 (0.0)	0 (0.0)	
Economic level					0.407
High	3 (1.6)	2 (1.5)	0 (0.0)	1 (3.7)	
Middle–high	40 (20.7)	23 (17.2)	8 (25.8)	9 (33.3)	
Middle	133 (68.9)	96 (71.1)	20 (64.5)	17 (63.0)	
Middle–low	15 (7.8)	12 (8.9)	3 (9.7)	0 (0.0)	
Low	2 (1.0)	2 (1.5)	0 (0.0)	0 (0.0)	
Education level					
Father ≥ College	150 (77.7)	109 (80.7)	20 (64.5)	21 (77.8)	0.147
≤High school	43 (22.3)	26 (19.3)	11 (35.5)	6 (22.2)	
Mother ≥ College	148 (76.7)	107 (79.3)	20 (64.5)	21 (77.8)	0.214
≤High school	45 (23.3)	27 (20.7)	11 (35.5)	6 (22.2)	

The data are presented as numbers (%). *p*-Value determined by analysis of χ^2^ test (categorical variables).

**Table 3:** Body image recognition and parents’ health status according to obesity status.

Variables	Total (*n* = 193)	Normal (*n* = 135)	Overweight (*n* = 31)	Obesity (*n* = 27)	*p*-Value
Awareness of body shape (judgment of child)					<0.001
Very thin	9 (4.7)	9 (6.7)	0 (0.0)	0 (0.0)	
Thin	20 (10.4)	20 (14.8)	0 (0.0)	0 (0.0)	
Moderate	74 (38.3)	66 (48.9)	4 (12.9)	4 (14.8)	
Fatty	75 (38.9)	36 (26.7)	22 (71.0)	17 (63.0)	
Very fatty	15 (7.8)	4 (3.0)	5 (16.1)	6 (22.2)	
Awareness of body shape (judgment of parents)					<0.001
Very thin	9 (4.7)	9 (6.7)	0 (0.0)	0 (0.0)	
Thin	25 (13.0)	25 (18.5)	0 (0.0)	0 (0.0)	
Moderate	75 (38.9)	70 (51.9)	4 (12.9)	1 (3.7)	
Fatty	64 (33.2)	28 (20.7)	19 (61.3)	17 (63.0)	
Very fatty	20 (10.4)	3 (2.2)	8 (25.8)	9 (33.3)	
Judgement matching the state of awareness of the body shape between child and parents					
Agreement	61 (31.6)	41 (30.4)	12 (38.7)	8 (29.6)	0.496
Disagreement	132 (67.4)	94 (69.6)	19 (61.3)	19 (70.4)	
Satisfaction of body shape (judgment of child)					<0.001
Very satisfied	36 (18.7)	31 (23.0)	4 (12.9)	1 (3.7)	
Satisfied	51 (27.5)	46 (34.1)	3 (9.7)	4 (14.8)	
Moderate	51 (26.4)	33 (24.4)	9 (29.0)	9 (33.3)	
Not satisfied	41 (21.2)	22 (16.3)	11 (35.5)	8 (29.6)	
Not satisfied at all	12 (6.2)	3 (2.2)	4 (12.9)	5 (18.5)	
Satisfaction of body shape (judgment of parents)					<0.001
Very satisfied	35 (18.1)	33 (24.4)	1 (3.2)	1 (3.7)	
Satisfied	42 (21.8)	41 (30.4)	0 (0.0)	1 (3.7)	
Moderate	47 (24.4)	33 (24.4)	8 (25.8)	6 (22.2)	
Not satisfied	49 (25.4)	22 (16.3)	17 (54.8)	10 (37.0)	
Not satisfied at all	20 (10.4)	6 (4.4)	5 (16.1)	9 (33.3)	
Judgement matching the state of awareness for the body shape between child and parents					
Agreement	85 (44.0)	63 (46.7)	13 (41.2)	9 (29.0)	0.004
Disagreement	108 (56.0)	72 (53.3)	18 (58.1)	18 (66.7)	
Father’s BMI	25.3 ± 3.0	24.9 ± 3.0	26.0 ± 2.8	26.4 ± 2.9	0.014 ^a^
Normal	34 (17.6)	30 (22.2)	3 (9.7)	1 (3.7)	0.064
Overweight	45 (23.3)	32 (23.7)	5 (16.1)	8 (29.6)	
Obesity	114 (59.1)	72 (54.1)	23 (74.2)	19 (66.7)	
Chronic disease in father	27 (14.0)	17 (12.6)	4 (12.9)	6 (22.2)	0.413
Mother’s BMI	22.8 ± 2.8	22.0 ± 2.7	25.0 ± 3.6	23.6 ± 2.7	<0.001 ^a,b^
Normal	102 (52.8)	82 (60.7)	9 (29.0)	11 (40.7)	<0.001
Overweight	37 (19.2)	28 (20.7)	5 (16.1)	4 (14.8)	
Obesity	54 (28.0)	25 (18.5)	17 (54.8)	12 (44.4)	
Chronic disease in mother	9 (4.7)	5 (3.7)	2 (6.5)	2 (7.4)	0.647
Obesity in both father and mother	35 (18.1)	17 (12.6)	13 (41.9)	5 (18.5)	0.001

BMI, body mass index. The data are presented as the mean ± standard deviation or numbers (%). *p*-Value determined by analysis of variance test (continuous variables) or χ^2^ test (categorical variables). ^a^ There existed a significant difference between the normal and obesity groups. ^b^ There existed a significant difference between the normal and overweight groups.

**Table 4:** Scores for nutrition quotients (NQs) and their factors according to obesity status.

Variables	Total (*n* = 193)	Normal (*n* = 135)	Overweight (*n* = 31)	Obesity (*n* = 27)	*p*-Value
Nutrition Quotient	60.3 ± 6.6	60.8 ± 6.6	62.7 ± 6.0	55.2 ± 4.4	<0.001 ^a,c^
Balance	59.6 ± 4.9	59.9 ± 4.4	61.0 ± 4.6	56.2 ± 6.4	0.003 ^a,c^
Diversity	57.1 ± 6.0	57.8 ± 6.0	56.4 ± 5.2	54.2 ± 6.2	0.002 ^a^
Moderation	45.9 ± 3.7	46.3 ± 3.5	47.2 ± 2.9	42.5 ± 3.6	<0.001 ^a,c^
Regularity	68.3 ± 5.6	69.3 ± 4.9	69.9 ± 4.8	61.3 ± 4.6	<0.001 ^a,c^
Practice	57.4 ± 3.7	57.9 ± 3.4	58.5 ± 3.3	53.5 ± 3.0	<0.001 ^a,c^

The data are presented as the mean ± standard deviation. *p*-Value determined by analysis of variance test (continuous variables). ^a^ There existed significant differences between the normal and obesity groups. ^c^ There existed significant differences between the overweight and obesity groups.

**Table 5:** Multivariate logistic regressions of an association between various factors and obesity status.

Variables	OR	95% CI ^1^	*p*-Value
Awareness of body shape			
(judgment of child)			
Very thin	0.81	0.71–1.09	0.284
Thin	0.90	0.69–1.05	0.169
Moderate	1.00		
Fatty	1.60	0.98–2.52	0.037
Very fatty	2.13	1.44–2.88	<0.001
Awareness of body shape			
(judgment of parents)			
Very thin	0.87	0.62–1.20	0.306
Thin	0.97	0.70–1.34	0.292
Moderate	1.00	1.00	
Fatty	2.04	1.01–2.49	0.008
Very fatty	2.21	1.56–3.23	0.001
Satisfaction of body shape			
(judgment of child)			
Very satisfied	0.84	0.60–1.28	0.289
Satisfied	0.93	0.82–1.30	0.376
Moderate	1.00	1.00	
Not satisfied	1.23	0.94–2.84	0.206
Not satisfied at all	2.28	1.27–3.01	0.005
Satisfaction of body shape			
(judgment of parents)			
Very satisfied	0.74	0.63–0.99	0.175
Satisfied	0.61	0.55–1.08	0.240
Moderate	1.00	1.00	
Not satisfied	1.22	0.99–1.62	0.045
Not satisfied at all	2.01	1.54–2.33	0.001
Father’s obesity status			
Normal	1.00	1.00	
Overweight	1.67	1.42–2.20	0.002
Obesity	2.53	2.01–3.65	<0.001
Mother’s obesity status			
Normal	1.00	1.00	
Overweight	1.21	0.93–1.84	0.042
Obesity	1.86	1.35–2.29	0.001
Nutrition Quotient (NQ)	0.72	0.51–0.90	0.007
Balance	0.85	0.62–0.94	0.180
Diversity	0.94	0.90–1.01	0.026
Moderation	0.61	0.52–0.98	<0.001
Regularity	0.75	0.49–1.13	0.204
Practice	0.63	0.51–0.82	<0.001

OR, odds ratio; CI, confidence interval. *p*-Values were obtained from logistic regression. ^1^ Model: adjusted for age, sex, current family composition, numbers of siblings, economic level, and education level.
